# Spontaneous and Excellent Healing of Bilateral Brown Tumors in Mandible after Endocrinal Therapy and Subtotal Parathyroidectomy: Case Report with 4-Year Follow-Up

**DOI:** 10.1155/2018/8070131

**Published:** 2018-07-02

**Authors:** Turker Yucesoy, Erdem Kilic, Fatma Dogruel, Fahri Bayram, Alper Alkan, Alper Celal Akcan, Figen Ozturk

**Affiliations:** ^1^Oral and Maxillofacial Surgery Department, Dentistry Faculty, Bezmialem Vakif University, Istanbul, Turkey; ^2^Oral and Maxillofacial Surgery Department, Dentistry Faculty, Erciyes University, Kayseri, Turkey; ^3^Endocrinology Department, Medicine Faculty, Erciyes University, Kayseri, Turkey; ^4^General Surgery Department, Medicine Faculty, Erciyes University, Kayseri, Turkey; ^5^Pathology Department, Medicine Faculty, Erciyes University, Kayseri, Turkey

## Abstract

Primary hyperparathyroidism is an endocrine disorder occurring due to increased secretion of parathormone resulting in a complex of clinical, anatomical, and biochemical alterations. On the other hand, excision of a parathyroid adenoma can normalize the metabolic status. A 24-year-old man was referred to the hospital with bilateral swelling and spontaneous gingival bleeding from posterior of the mandible also with radiolucent well-demarcated lesions bilaterally in the mandibular third molar regions. After consultations, the patient was hospitalized in the endocrinology department where further tests were performed due to highly increased PTH level as 714 pg/ml. Bilateral brown tumors started to regress spontaneously, and no additional surgery was required after subtotal parathyroidectomy was performed. The presented case is the first patient whose bilateral brown tumors in the jaws spontaneously and totally healed after subtotal parathyroidectomy and endocrinal therapy who was strictly followed up for 4 years even though the lesions were associated with impacted third molars.

## 1. Introduction

The giant cell lesions associated with primary hyperparathyroidism (PHPT) are referred to as brown tumors in the jaws. PHPT is an endocrine disorder occurring due to increased secretion of parathormone resulting in a complex of clinical, anatomical, and biochemical alterations. Parathormone (PTH) controls the calcium (Ca) level in the plasma and extracellular fluid. Increased production of PTH usually causes high levels of serum Ca and alkaline phosphatase (ALP) levels while P levels are decreased [1].

Increased PTH level can cause an imbalance of osteoblastic and osteoclastic activities. This imbalance is characterized by the resorption of the bone, leaving sinusoidal vascular spaces and fibrous connective tissue [2]. One of the skeletal lesions observed in PHPT is the brown tumor also termed as Von Recklinghausen's disease of the bone or osteitis cystica fibrosa. Brown tumors have been associated with primary hyperparathyroidism which is mostly asymptomatic; a painful exophytic mass may be observed. Radiographically, it appears as a unilocular or multilocular lesion with an irregular periphery. Histologically, it is a focal giant cell lesion which demonstrates multinucleated giant cells within a fibrovascular stroma admixed with areas of hemorrhage and hemosiderin deposits [3].

Histological findings include dilated blood vessels, fibromuscular proliferation, and macrophages. Deposits of hemosiderin cause the “brown” appearance of this tumor [4]. HPT is categorized into 4 types: primary HPT is caused by parathyroid adenomas (85%), hyperplasias (10%), and carcinomas (5%); secondary HPT occurs as a compensatory increase in parathormone levels due to hypocalcemia or vitamin D deficiency; tertiary HPT presents in patients with long-standing secondary HPT resulting in autonomous functioning of parathyroid gland; the fourth type is an ectopic variant seen in patients with other malignancies [5]. PHPT is sporadic in the vast majority of the cases. However, it is essential for the surgeon managing PHPT to know that it may occur in familial settings. The commonly associated conditions are MEN (multiple endocrinal neoplasias) syndromes (MEN1, MEN2A), FIHP (familial isolated hyperparathyroidism), HPT-jaw tumor syndrome, autosomal dominant mild hyperparathyroidism (ADMH), and neonatal severe hyperparathyroidism (NSHPT) [6].

After the endocrine treatment, these lesions can regress spontaneously in other bones [7, 8], but to our knowledge, the presented case is the first patient whose bilateral brown tumors in the jaws spontaneously and totally healed after endocrinal therapy and who was followed up for 4 years.

## 2. Case Report

**Figure 1:**
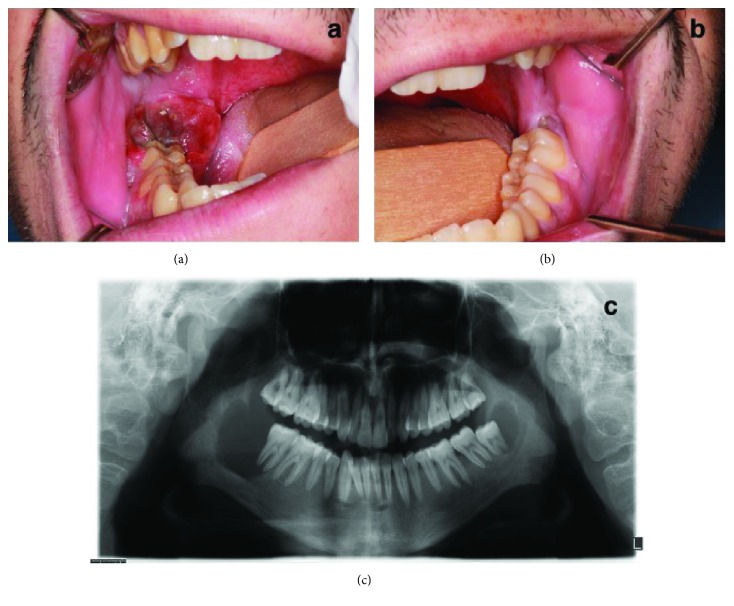
(a) Initial intraoral appearance of the brown tumor (right side). (b) Initial intraoral appearance of the brown tumor (left side). (c) Diagnostic panoramic radiography of bilateral brown tumors.

A 24-year-old man was referred to Erciyes University Faculty of Dentistry, Oral and Maxillofacial Surgery Department Clinic, Kayseri, Turkey, with bilateral swelling and spontaneous gingival bleeding from the posterior of the mandible. His medical history was noncontributory. There was no visible swelling, tenderness, or pus discharge. Skin color and temperature were normal. Intraoral examination revealed pericoronitis and spontaneous bleeding from the periodontal pocket of the right mandibular second molar and swelling in the bilateral retromolar regions (Figures 1(a) and 1(b)). In the radiographic examination, bilateral not well-demarcated radiolucent lesions in the posterior regions of the mandible, measuring 4 × 3 × 3 cm on the right and 2.5 × 1.5 × 1.5 cm on the left, were observed (Figure 1(c)).

After questioning the patient's family history, the patient stated that his father had a serious endocrinal disease 30 years ago and he received endocrine treatment because of a problem in his parathyroid glands. Therefore, we suspected of brown tumor for the presented case, because of his family's history of endocrine disorders and the panoramic radiography, so the patient was offered to receive some specific blood tests. Biochemical tests demonstrated extremely high PTH level and high level of serum Ca (12.8 mg/dl) and ALP (220 U/L). PTH level was 714 pg/ml which was conspicuously higher from the normal levels (15–65 pg/ml).

After consulting with Erciyes University Medicine Faculty Endocrinology Department, the patient was hospitalized in the endocrinology clinic and further tests were performed. As mentioned earlier, because of the familial tendency of the patient and hyperplasia in the parathyroid gland, endocrinologists suspected of MEN syndrome. For that, the patient and some relatives received several examinations and genetic tests for MEN syndrome but the results were negative for MEN. So the endocrinologists consulted the patient to the General Surgery Department in Erciyes University Medicine Faculty for surgical treatment of hyperplastic parathyroid gland. After radiographical and clinical examinations by general surgeons, parathyroidectomy was decided to perform a surgical procedure as soon as possible. The general surgeons gained access to the thyroid gland under general anesthesia. After mobilizing of the left thyroid lobe, parathyroid glands were exposed with measurements: 4 cm in the inferior pole and 2 cm in the superior pole where the glands were measured as 3.5 cm in the superior pole and 2 cm in the inferior pole after mobilization of the right thyroid lobe, respectively. The surgeons performed subtotal parathyroidectomy for the lesions. All the pathological glands were removed, but only one small portion of the right inferior lobe was remained and signed with metallic clips. The recurrent laryngeal nerves were protected during the operation.

**Figure 2:**
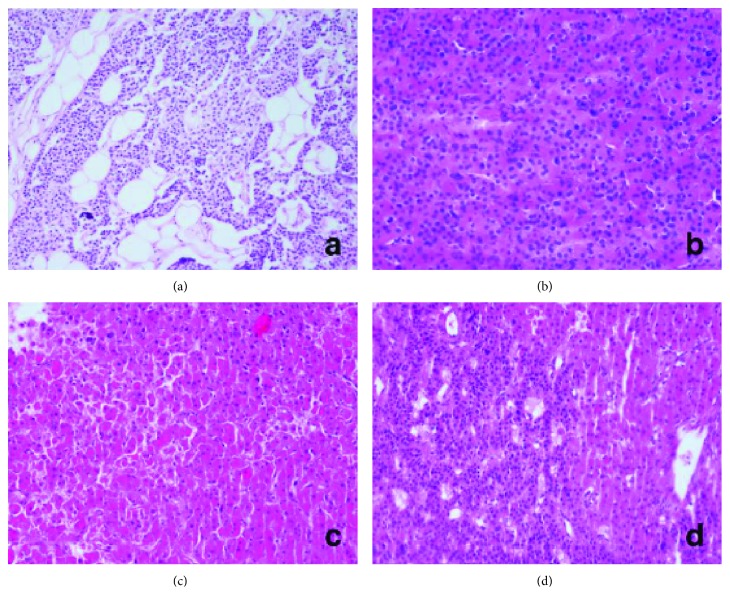
Microscopic finding from the parathyroid glands. (a) Right inferior paratiroid hyperplasia (H&E stain). (b) Right superior paratiroid hyperplasia (H&E stain). (c) Left inferior paratiroid hyperplasia (H&E stain). (d) Left superior paratiroid hyperplasia (H&E stain).

Four different-sized macroscopic biopsy specimens from parathyroid glands were sent to the Pathology Department of the Erciyes University Medicine Faculty. According to the results, no adipose tissue was observed in the whole specimen which was covered with a thin fibrous capsule macroscopically. Mostly, main cells of parathyroid gland but rarely oxyphilic cells were observed in the tissue. The cells showed a diffuse lining; however, trabecular or acinar structures were also seen. None of those atypical tissues were in the neighborhood of a normal parathyroid tissue. Some of the giant cells had a hyperchromatic nucleus. As a result, parathyroid hyperplasia was diagnosed pathologically because of morphological and clinical evidence (Figure 2).

**Figure 3:**
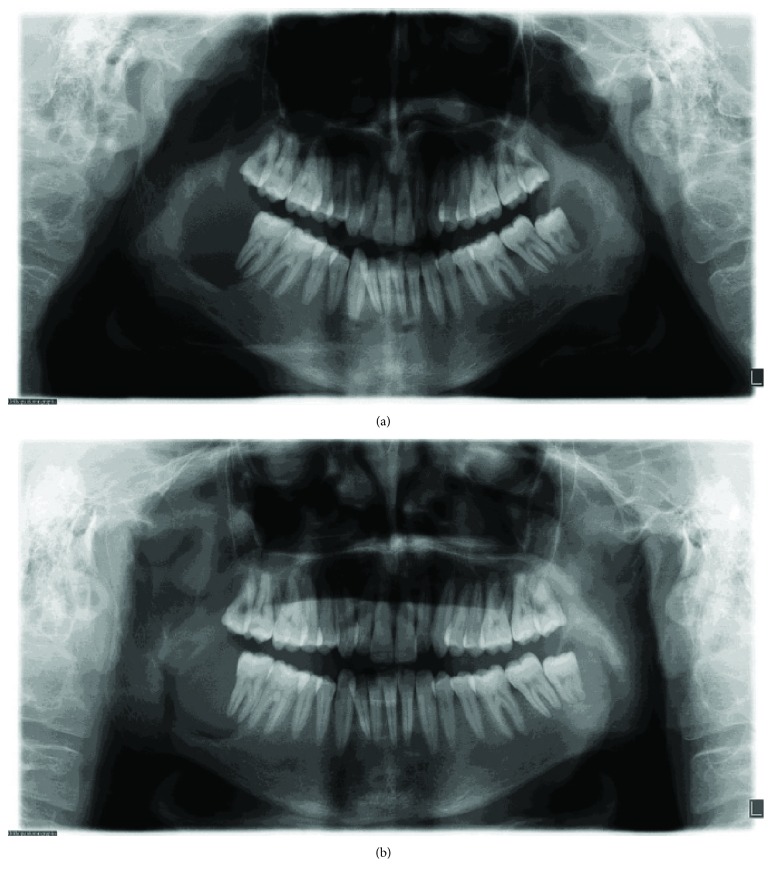
(a) Three months of follow-up: panoramic radiography of bilateral brown tumors. Notice to decreased radiolucency for the brown tumor in the left side of the mandible. (b) First year of follow up: panoramic radiography of bilateral brown tumors. Notice to decreased radiopacity for the brown tumor in the bilateral sides of the mandible.

After surgical treatment of parathyroid glands and successful endocrinal therapy by Erciyes University Medicine Faculty, the patient was hospitalized for almost 3 months. After being discharged from the hospital, the patient applied to our clinic for his follow-ups and bilateral regression of the lesions on the left side of mandible occurred spontaneously (Figures 3(a) and 3(b)).

However, even though lesion-related molars were indicated for extraction as soon as possible, the patient insisted that he did now want any surgical procedures because he felt no disturbance at all. Because the patient rejected all the alternative treatments for his lesions in the mandible, frequent follow-ups were considered for him and we did it for 4 years.

**Figure 4:**
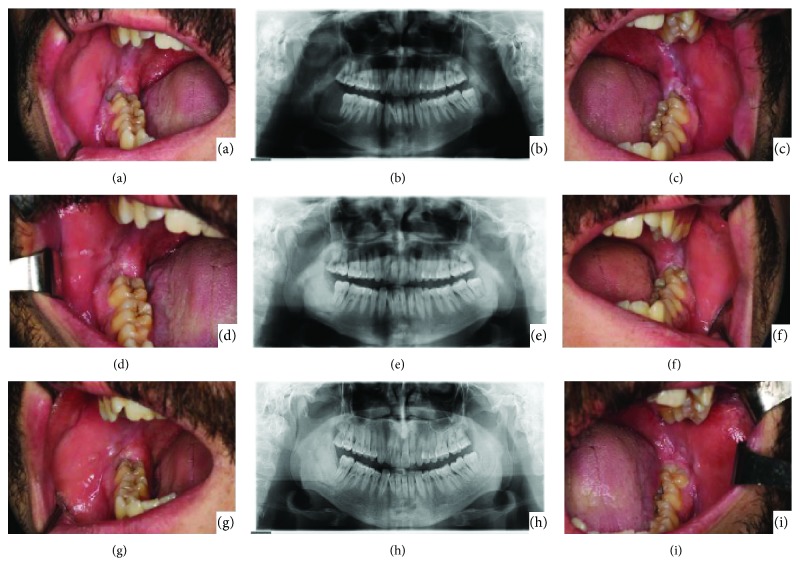
(a) Six months of follow-up: panoramic radiography of bilateral brown tumors. Notice to decreased radiolucency for the brown tumor in the bilateral sides of the mandible. (b) Six months of follow-up: intraoral appearance of the brown tumor (right side). (c) Six months of follow-up: intraoral appearance of the brown tumor (left side). (d) Second year of follow-up: panoramic radiography of bilateral brown tumors. Notice not to observe recurrence for brown tumors in the bilateral sides of the mandible. (e) Second year of follow-up: intraoral appearance of the brown tumor (right side). (f) Second year of follow-up: intraoral appearance of the brown tumor (left side). (g) Fourth year of follow-up: panoramic radiography of bilateral brown tumors. (h) Fourth year of follow-up: intraoral appearance of the brown tumor (right side). (i) Fourth year of follow-up: intraoral appearance of the brown tumor (left side). Notice to the left mandibular third molar eruption is almost completed.

In the radiographic examination, partial calcification of the lesions was observed after 6 months (Figure 4(a)). Also, after intraoral examination, spontaneous periodontal healing was uneventful (Figures 4(b) and 4(c)). Radiological and intraoral examinations were performed carefully in follow-ups for first and second years. (Figures 4(d)–4(f)). There was no evidence of recurrence at a 4-year follow-up, either radiographically or intraorally (Figures 4(g)–4(i)).

## 3. Discussion

Brown tumors arise in the jaws and they may be misdiagnosed with giant cell tumors, giant cell reparative granuloma, and cherubism. Because it is difficult to distinguish brown tumor from other giant cell lesions histopathologically, the clinical diagnosis should be made based on the association with HPT [7].

Radiographically, the lesions appear as well-demarcated monocular or multilocular osteolytic radiolucencies resembling cystic lesions. Other radiographic findings are the loss of lamina dura surrounding roots of teeth and reduced bone density [9, 10]. Our patient had similar radiographic features as mentioned previously (Figure 1).

The diagnosis of a brown tumor can be made through the use of biochemical tests such as serum calcium, alkaline phosphatase (ALP), phosphorus (P), sodium (Na), potassium (K), calcium (Ca), and parathyroid hormone (PTH) levels in the presence of giant cell lesions [11]. Uhluhizarci et al. revealed that complicated hyperparathyroidism is an important health problem in our region, and primary hyperparathyroidism should be kept in mind in all patients with bone and joint complaints [12]. However, hyperparathyroidism is discovered coincidentally on routine biochemical and radiological investigations [13].

Ameloblastoma, osteomyelitis, and odontogenic cysts are considered for differential diagnosis of these tumors [14, 15]. Mostly, brown tumor is a sporadic disease but may also occur in a familial pattern as an autosomal dominant condition like hyperparathyroidism-jaw tumor syndrome (HPT-JT syndrome) and multiple endocrine neoplasia (MEN) syndrome [10]. About 1 in 10 cases of primary hyperparathyroidism is hereditary, occurring as an isolated form or associated with other abnormalities. If sporadic, individuals should be advised that there is a nonnegligible recurrence risk since there is a small but significant genetic component to this multifactorial disease but it can be difficult to exclude a hereditary syndrome [16]. For this reason, not only our patients but also some of his relatives were examined very carefully by the Endocrinology Department of Erciyes University after our patient's blood test revealed extremely high PTH level as 714 pg/ml and serum calcium was 12.8 mg/dl. But even though the genetical results were negative for MEN syndrome, the patient and the family were also recommended to be controlled for life-long time in the endocrinology department to avoid recurrence.

There are many options for treatment of the brown tumor. Before the medical or surgical treatment of the lytic bone lesions, hormonal regulation is necessary. Bony lesions tend to regress spontaneously if the calcium and PTH levels normalize [9]. Silverman et al. [2] reported that excising a brown tumor was not necessary when HPT resolved whereas Steinbach et al. [17] reported that brown tumors could be treated by local radiotherapy or curettage. However, many authors have reported that they resected any remaining brown tumors after HPT was resolved [18–21].

The surgical treatment for a brown tumor in the jaws includes enucleation and curettage and radical resection and reconstruction [4, 22]. Some authors initially treat this lesion with systemic or intralesional corticosteroids and when size was reduced, then surgical excision can be performed [21]. However, some authors reported that the osteitis fibrosa cyctica in the bone is a kind of PTH-related tumor which is possible to heal spontaneously [7, 8]. In the present case, although it is widely spread into the posterior mandible in the right side and possible to be the same on the left side, no surgical procedure was needed after endocrine therapy because of patient's tumors in both sides of the mandible reduced instantly during the follow-ups.

In the first year of his follow-up, we also were expecting to have a decrease in radiolucency and perform a more conservative surgery, but surprisingly, brown tumors in both sides were totally healed uneventfully. With the coordination of his endocrinal therapy controls, radiographical and oral examinations were also done in Erciyes University, Dentistry Faculty for 4 years as shown in Figure 4.

Because it is difficult to distinguish brown tumor from other giant cell lesions histopathologically, a clinical decision should be made with considering the underlying systemic event. In their study, Guney et al. aimed to remind endocrinologists and maxillofacial surgeons that these tumors have to be taken into account [23]. The clinicians should question their patients' medical and family history very carefully not to miss any clue for the diagnosis and proper treatment approach.

We report an unusual case of brown tumor in the mandible that completely recovered spontaneously after endocrine therapy. Thankfully, to the follow-ups which we performed carefully and patiently, the second molar in the right mandible was protected and the extraction of this tooth was not needed. Also, we want to encourage the clinicians not to perform the surgery so emergently and recommend them to follow their patients as long as possible for further tests or surgeries, because surgical excision of a brown tumor is not always necessary if the endocrinal disorder is treated and controlled properly via normalizing the PHPT markers.
